# The Role of Heparin and Glycocalyx in Blood–Brain Barrier Dysfunction

**DOI:** 10.3389/fimmu.2021.754141

**Published:** 2021-12-21

**Authors:** Rui Yang, Mingming Chen, Jiayin Zheng, Xin Li, Xiaojuan Zhang

**Affiliations:** Department of Critical Care Medicine, The First Affiliated Hospital of China Medical University, Shenyang, China

**Keywords:** blood-brain barrier, endothelial cell, glycocalyx, heparin, dysfunction, neurological disease

## Abstract

The blood-brain barrier (BBB) functions as a dynamic boundary that protects the central nervous system from blood and plays an important role in maintaining the homeostasis of the brain. Dysfunction of the BBB is a pathophysiological characteristic of multiple neurologic diseases. Glycocalyx covers the luminal side of vascular endothelial cells(ECs). Damage of glycocalyx leads to disruption of the BBB, while inhibiting glycocalyx degradation maintains BBB integrity. Heparin has been recognized as an anticoagulant and it protects endothelial glycocalyx from destruction. In this review, we summarize the role of glycocalyx in BBB formation and the therapeutic potency of heparin to provide a theoretical basis for the treatment of neurological diseases related to BBB breakdown.

## Introduction

The blood-brain barrier (BBB) acts as a blockade to prevent harmful substances from entering the brain and thus protects the normal operation of cerebral cells. This protective function depends on the unique and dynamic structure of the BBB, which is composed of capillary endothelial cells with tight junction, basement membrane and astrocytic endfeet (1, 2). Damage to this structure leads to the dysfunction of the BBB and subsequent disorder of brain activity, which has been demonstrated in various neurological diseases (3, 4).

Glycocalyx refers to glycoproteins and proteoglycans that covers the luminal surface of endothelial cells with a gel-like characteristic. This special structure contributes to the formation of a physical and charged barrier (5–8) and mediates mechanical transduction (9, 10), vascular permeability and the inflammatory response (11, 12). The role of this proteoglycan coat in activating antithrombin has been well demonstrated. Dysfunction of the BBB has been associated with aberrations in glycocalyx generation and function.

Heparin is a strongly acidic polysulfated mucopolysaccharide that was named based on its first identification in liver tissue. It also resides in tissues such as the lung, vascular wall and intestinal mucosa as a natural anticoagulant. Heparin protects endothelial glycocalyx from degradation and thus shows efficacy in mitigating cerebral cell impairment and improving prognosis in diseases with BBB dysfunction, such as subarachnoid hemorrhage (13, 14), traumatic brain injury (TBI) (15)and epilepsy (16). In this brief review, we summarize several recent studies on BBB derangement, focusing on the role of glycocalyx as well as the therapeutic value of heparin. We further discuss several hypothetical mechanisms underlying the benefits of these two regulators.

## BBB

The BBB is present between the brain and vascular tissues in the central nervous system, including penetrating arteries and arterioles, dense capillary beds, posterior capillary venules, and drainage venules (17). It is mainly composed of brain microvascular endothelial cells, pericytes, astrocytes and acellular components of the basement membrane (BM) (18, 19). The crucial utility of BBB in maintaining the environmental balance within the brain has been investigated in detail. Its protective function is achieved by inhibiting the entry of peripheral immune cells to brain tissue, delivering nutrients, removing toxic substances, and controlling the solute exchange between blood and brain. The BBB also works as a semi-permeable barrier to regulate the inflow and outflow of molecules (20, 21).

Nerve signaling within the central nervous system (CNS) requires a highly controlled microenvironment. BBB, Blood-CSF barrier and arachnoid barrier form barriers between the blood and CNS. The BBB at the level of cerebral microvascular endothelium is the main site of blood exchange in the central nervous system. All organisms with well-developed central nervous systems have a BBB (22). In the brain and spinal cord of mammals, including humans, the BBB is produced by ECs that form the walls of capillaries. The combined surface area of these microvessels constitutes by far the largest blood-brain exchange interface, with the total area used for exchange, in the average adult brain being between 12 and 18 square centimeters. CNS vessels are continuous, non-porous vessels, but also contain a range of additional properties that enable them to tightly regulate the movement of molecular ions and cells between the blood and CNS (17, 23). The severe restrictions on the barrier ability makes the BBB to strictly regulate the steady state of the central nervous system, the function and protect the central nervous system neurons in the normal from the effects of toxin pathogens inflammatory injury and disease is very important. The BBB is characterized primarily within ECs, but is induced and maintained through key interactions with parietal cells, immune cells, glial cells, and nerve cells that interact at the neurovascular unit.

ECs are connected by tight junctions (TJs), a key feature of the BBB that significantly reduces the penetration of polar solutes through paracellular diffusion channels between plasma and brain cell extracellular fluid *via* endothelial cells (24, 25). In addition, CNS ECs contain two types of transporters. The first type is efflux transporter, which uses ATP hydrolysis to transport a wide range of small molecules back to the blood along concentration gradient (26). The second is a highly specific nutrient transporter that promotes the passage of specific nutrients into the central nervous system through the blood-brain barrier, as well as the clearance of specific wastes from the central nervous system into the blood (27). The outer membrane surface of EC is covered by the basement membrane. These membrane surfaces merge around capillaries, but separate behind capillaries at venules, forming a perivascular space for cerebrospinal fluid drainage for immunosurveillance (28). Pericytes play an important role in regulating angiogenesis, extracellular matrix deposition, wound healing, immune cell infiltration, and blood flow induced by neural activity, which locate outside the ECs and embed in the vascular basement membrane (29). Moreover, the BBB also contains Astrocytes. Astrocytes are a major glial cell type that tends to polarize cellular processes surrounding neuronal processes or blood vessels (30). The ends of the basal processes almost completely surround blood vessels and contain a discrete set of proteins including dystroclycan, dystrophin, and aquaporin 4. This astrocyte endothelial interaction is critical in regulating blood flow (31). Finally, CNS-associated macrophages are elongated cells located between astrocyte terminals and parenchymal blood vessels (mainly arteries and veins), and when they are inactive they extend along the perivascular space and provide the first line of natural immunity by phagocytic debris (32, 33).

## BBB Dysfunction

Breakdown of the BBB occurs in a variety of neurological diseases, such as multiple sclerosis (MS), stroke, Alzheimer’s disease, vascular dementia, cerebral microvascular disease, brain trauma and epilepsy (34–43). BBB disruption leads to ion dysplasia edema and neuroinflammation, leading to neuronal dysfunction increased intracranial pressure and neuronal degeneration. However, the mechanisms of BBB dysfunction and their role in disease onset and progression or recovery are not fully understood. The term BBB breakdown conjudes up images of physical walls being broken down, allowing molecules to flow continuously from the blood into the brain. However, the BBB is not a wall, but a set of physiological properties, and changes are just one property (transcytosis transport) that can significantly alter the neural environment (44). Various mechanisms of BBB dysfunction cause different characteristics of central system diseases. Therefore, the BBB is not a switch, and it is crucial to understand the characteristics and consequences behind each instance of dysfunction.

## Multiple Sclerosis (MS)

Central nervous system immune infiltration is a key step in the pathophysiology of MS. The primary sites of central nervous system immune monitoring in healthy persons are the blood CSF barriers of the choroid plexus and meninges, both of which are important sites of initial lymphocyte activation in experimental autoimmune encephalomyelitis (EAE) model (45–51). These immune cells first enter the perivascular space around posterior venules of capillaries (52), and enter the parenchyma after decomposition of basement membrane (53, 54). Leukocyte derived cytokines activate CNS ECs and induce expression of leukocyte adhesion molecules (50, 55, 56), resulting in a large number of immune cell infiltrates into the parenchyma. Limiting immune cell transport across the BBB has been shown to be effective against MS through preventing immune cells from interacting with endothelial VCAM1, greatly reducing the formation of new lesions (57). The time course of leakage was studied by dynamic magnetic resonance enhancement (58–61). Although barrier leakage is almost always present in new lesions, it is rarely observed in older lesions (58, 59). Interestingly, MRI evidence suggests that BBB permeability is the initial event for the formation of lesion subsets, but in other cases, lesion formation precedes barrier dysfunction (60).

## Ischemia/Stroke

There are two stages of BBB dysfunction in stroke: increased nonspecific molecular endocytosis is the first stage of dysfunction, followed by structural changes in tight junctions (62). The leakage was evident in the first few hours after the initial injury, then decreased, and then reappeared the next day (63, 64). Most cell death resulting in neurological impairment occurs within a few days of stroke. Therefore, secondary BBB leakage may be an important therapeutic target. It also been reported to reduce infarct volume by leukocyte adhesion molecule knockout or antibodies to leukocyte adhesion molecules (65–68). So the importance of leukocyte infiltration in pathogenesis remains questionable.

## Epilepsy

There is a clear link between epilepsy and BBB dysfunction. Experimental disruption of the BBB by osmotic shock can lead to epileptic seizures in patients (69), while diseases with impaired BBB such as infectious inflammatory stroke and traumatic brain injury also can lead to epileptic seizures and seizures (70). In addition, neuroinflammation has been speculated to be related to the etiology of epilepsy. The onset and recurrence of epilepsy can be inhibited by pharmacological or gene knockout blocking leukocyte vascular interactions (71). Interestingly, bbB-GLUT1-deficient patients develop epilepsy (72, 73), demonstrated the critical role of BBB transport in normal brain function. Brain tissue analysis of epileptic patients showed increased parenchymal albumin, suggesting large molecules of blood-brain extravasation (74, 75). At the visual level, there is also evidence that blood-brain barrier leakage can be seen on MRI enhancement in epileptic patients (76–78). In addition, patient samples showed a regional reduction of GLUT1 (79), and positron emission tomography showed reduced uptake and metabolism of epileptic foci (74, 80).

## Alzheimer’s Disease (AD)

Cerebrovascular endothelial dysfunction and white blood cells crossing the BBB may be involved in the occurrence and development of neurodegenerative diseases such as AD and Parkinson’s disease (PD). Some imaging studies have found evidence of BBB leakage in AD patients and suggested that BBB dysfunction is an early biomarker of AD (4, 81–83). In addition to leakage, BBB Aβ transport dysfunction may also lead to pathological changes in AD (84, 85). Late glycation end-product receptor (RAGE) inputs Aβ into the central nervous system (86). RAGE activity was hypothesized to drive CNS amyloid deposition in AD patients (87). In contrast, soluble LRP and ApoE are both Aβ chaperones on the cell surface, which are associated with the clearance of receptors and promote Aβ extrusion from the brain back into the blood through the blood-brain barrier (88). In AD, these interstitial channels seem to be altered, which is hypothesized to lead to the accumulation of soluble Aβ in the perivascular space and the formation of toxic Aβ oligomers (89). Soluble amyloid B can also stimulate the metastasis of monocytes, enhance the pathology of Tau protein, induce the secretion of pro-inflammatory cytokines (TNF and IL-6) and chemokines, activate the activator MT1-MMP of MMP-2, stimulate the production of MMP-9, and activate the production of reactive oxygen species (ROS) (90). However, a recent study found that BBB dysfunction is an early marker of cognitive decline unrelated to Aβ or Tau accumulation (83), but more details are needed regarding the extent of BBB dysfunction at various points during the AD time course.

## Glycocalyx in BBB Integrity and Activity

Glycocalyx covers the surface of the lumen side of vascular endothelial cells. Glycocalyx is mainly composed of proteoglycan (PG) of varying sizes and the negatively charged glycosaminoglycan (GAG) side chain (43). The core proteins of glycocalyx bind to the cell membranes through transmembrane domains (syndecans) or glycosylphosphatidylinositol anchors (glypicans). GAG side chains are divided into five types: heparin sulfate (which accounts for 50%–90% of GAG side chains), chondroitin sulfate, hyaluronan, keratin sulfate and dermatan sulfate. Endothelial glycocalyx constitutes neurovascular units, an important physiological structure that guarantees neuronal homeostasis and the integrity of the vascular wall (91).

**Figure 1:**
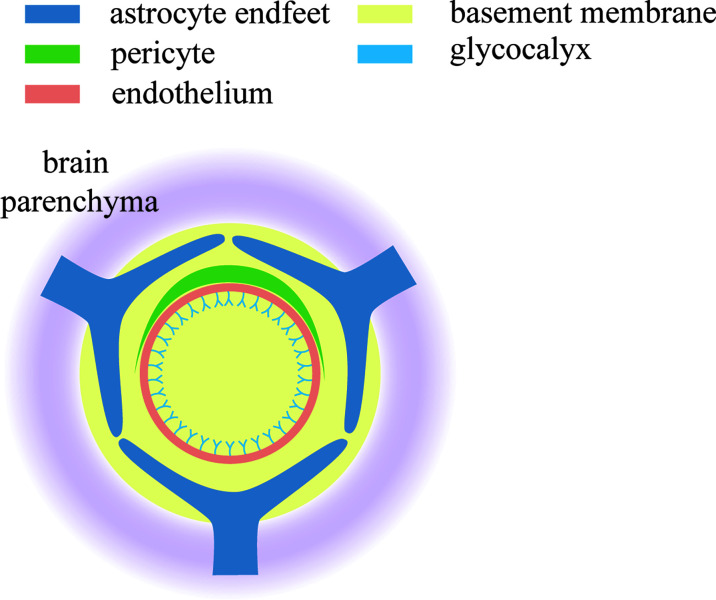
Components of the tripartite BBB. Glycocalyx is located on the endothelial surface of blood vessels and is in contact with various components in the blood. It is the first line of defense of BBB.

However, what exactly is the relationship between glycocalyx and BBB? Nikolay Kutuzov.et al used two-photon microscopy to document the passive transport of four different sizes of luciferin sodium (376 Da), Alexa Fluor (643 Da), 40 kda dextran and 150 kda dextran from the blood to the brain at the individual cortical capillary level in anesthetized mice. This experiment supports that the BBB consists of a calyx glycosus on the lumen side of the endodermis, the endothelium itself, and the extravascular lumen (92) (**Figure 1**).

Regarding the mechanisms underlying the protective function of glycocalyx in endothelial performance, the formation of a physical barrier between plasma and endothelial cells is the most critical factor as it reduces the chance for harmful circulatory components to contact the endothelial surface (93). A complete and stable endothelial glycocalyx inhibits the interaction between not only molecules but also blood cells and endothelial cells (94–96). The degradation of the endothelial glycocalyx during inflammation or ischemic disease enhances the interactions between blood cells and endothelial cells (95, 97), which causes endothelial dysfunction and damage to the BBB. Moreover, the physical barrier of the endothelial glycocalyx mitigates the oxidative stress–induced BBB dysorganization. As described above, the endothelial glycocalyx contains a charged side chain with a large number of sulfate residues, especially heparin sulfate. This feature confers endothelial glycocalyx a net negative charge, and thus it acts like a giant molecular sieve that resists negatively charged molecules. This kind of surface also forms an electrostatic barrier for plasma cells and proteins (8).

**Figure 2:**
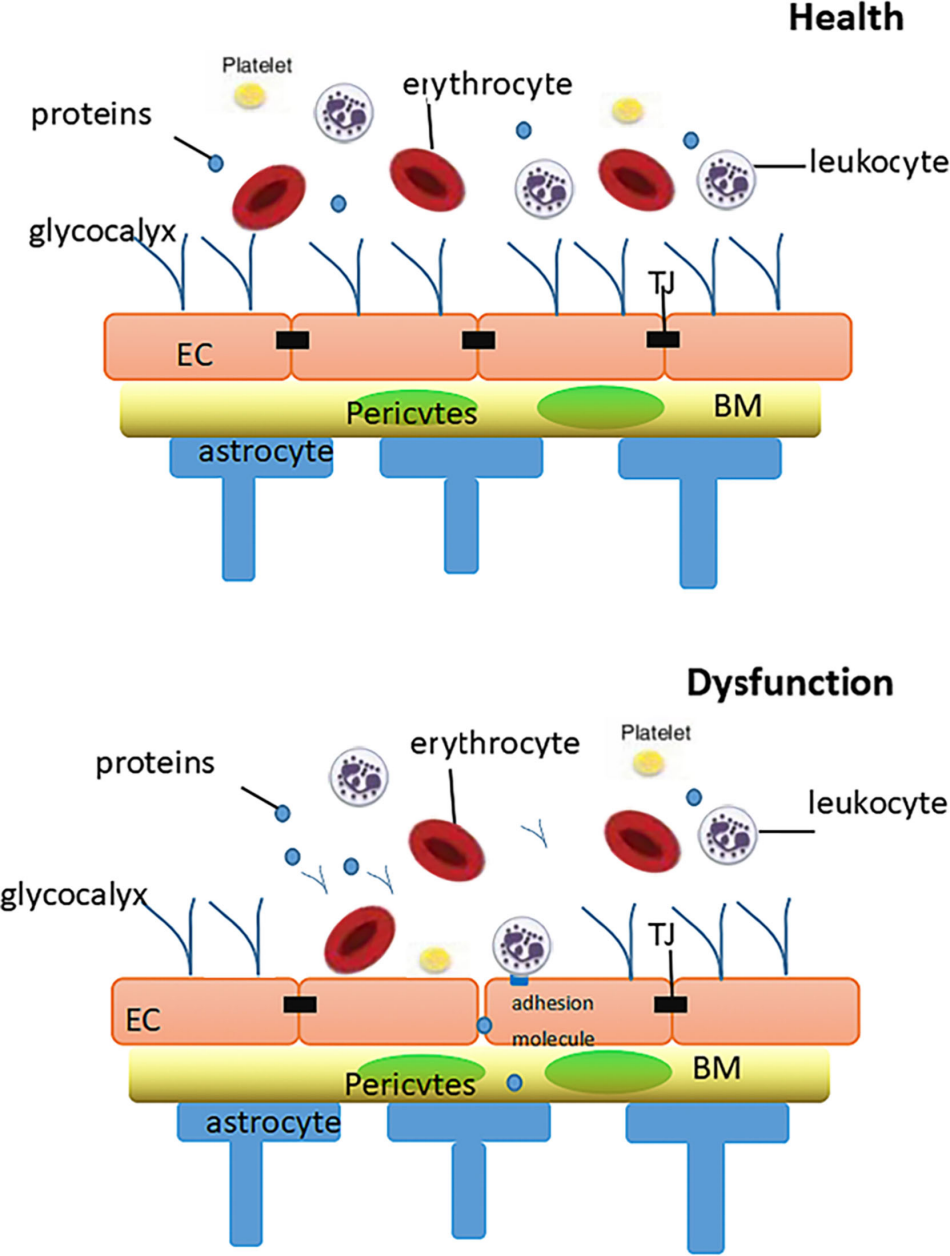
Diagram of the relationship between glycocalyx and the blood-brain barrier in health and dysfunctional states. In health: The intact glycocalyx can limit vascular wall permeability to macromolecules (including albumin).In dysfunction: When damage affects both the glycocalyx, increases in the vascular wall permeability will result, resulting in leukocyte adhesion and thrombosis. BM, basement membrane; TJ, tight junction; EC, endothelial cell.

Another trait of glycocalyx in regulating shear stress imposed on vascular endothelium has been uncovered. Some studies demonstrated the role of glycocalyx in mechanical transduction (9, 10) as its specific structure translates mechanical forces into biochemical signals, such as the activation of endothelial nitric oxide synthase and the formation of nitric oxide (NO) (98). Under normal physiological conditions, the adhesion molecules of endothelial cells, such as PECAM, VCAMs and ICAMs, are hidden in the structure of glycocalyx (11), which also prevents the interaction of platelets or leukocytes with endothelial cells. During inflammation, the endothelial glycocalyx is destroyed by TNF-α and LPS as well as activated mast cells, which release cytokines, proteases, histamine, and HPSE. Disruption of the glycocalyx exposes endothelial cells to adhesion molecules, triggering the rolling and adhesion of leukocytes and platelets (12) and leading to the coagulation of blood. In addition, antithrombin III blocks thrombin activation factor IX and activation factor X. This anticoagulant activity is enhanced by binding heparin sulfate. Moreover, heparin cofactor II is activated after the stimulation of GAG by dermatan sulphate. Thromboxane contains chondroitin sulfate, another GAG that interacts with thrombin to mobilize protein C anticoagulant pathways. Tissue factor pathway inhibitors depress the activity of VIIA and Xa by binding to heparin sulfate (11) (**Figure 2**).

Endothelial glycocalyx coats healthy vascular endothelium and plays an important role in vascular homeostasis. Although cerebral capillaries are categorized as continuous, as are those in the heart and lung, they likely have specific features related to their function in the blood brain barrier (99). In a previous experiment, C57BL6 mouse brain hearts and lungs were treated with an alkaline Fxative containing lanthanum, which preserves the structure of glycocalyx and is examined by scanning and transmission electron microscopy. It found that endothelial glycocalyx is present over the entire luminal surface of cerebral capillaries. The percent area physically covered by glycocalyx within the lumen of cerebral capillaries was about 40%, which is significantly more than in cardiac and pulmonary capillaries. Upon lipopolysaccharide-induced vascular injury, the endothelial glycocalyx was reduced within cerebral capillaries, but substantial amounts remained. By contrast, cardiac and pulmonary capillaries became nearly devoid of glycocalyx. These findings suggested the denser structure of glycocalyx in the brain is associated with endothelial protection and may be an important component of the blood brain barrier (99). However, in contrast with glycocalyx on other blood vessels, the specificity of the protective mechanism of glycocalyx on BBB is not known.

**Figure 3:**
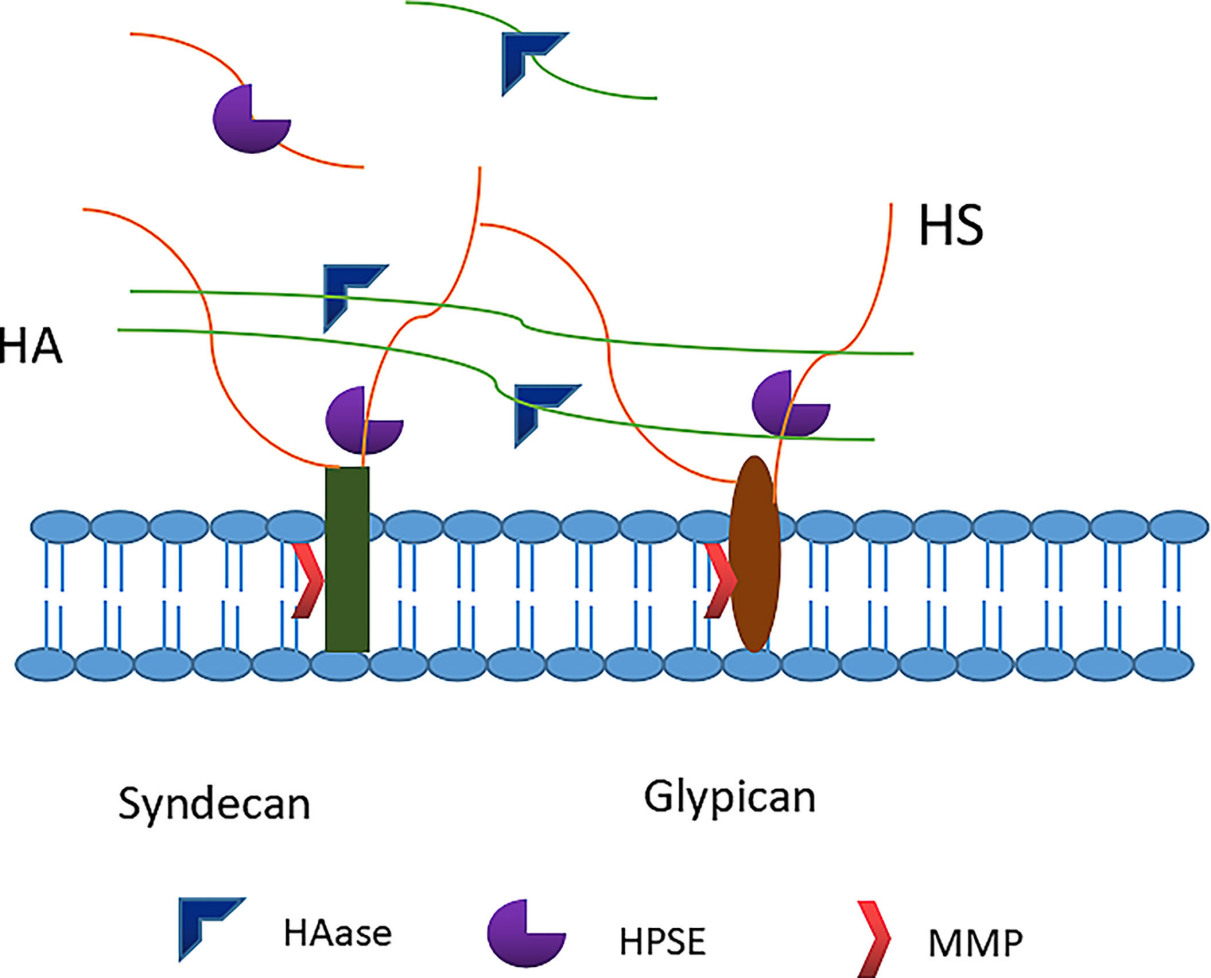
Degradation of glycocalyx. Heparanase directly cleaves the heparan sulfate chains attached to core proteoglycans. Matrix metalloproteinases (MMPs) cleave proteoglycans (e.g. syndecan-1) directly from the endothelial cell membrane. Hyaluronidase cleaves the hyaluronan. HS, heparan sulfate; HA, hyaluronic acid; HPSE, heparanase; HAase, hyaluronidase; MMP, matrix metalloproteinase.

As an important protector of BBB integrity (100), glycocalyx is fragile and highly susceptible to adverse episodes such as ischemia-reperfusion, inflammation, trauma, sepsis, and high blood volume (11, 101). Major constituents of the glycocalyx, including syndecans, heparan sulphates and hyaluronan, are shed from the endothelial surface into blood and urine in a variety of acute and chronic clinical conditions (102, 103). Matrix metalloproteases may shed syndecans and heparanase, released from activated mast cells, cleaves heparan sulphates from core proteins. According to new data, not only hyaluronidase but also the serine proteases thrombin, elastase, proteinase 3 and plasminogen, as well as cathepsin B lead to loss of hyaluronan from the endothelial surface layer, suggesting a wide array of potentially destructive conditions (102) (**Figure 3**). It is worth mentioning that there are four types of Syndecans (numbered 1 through 4), but Syndecan-1 (sdc1) appears to be the most common type of endovascular endothelial surface, usually sdc1 has been measured in human studies. Although in SDC1 (-/-) mice it can be replaced by other proteoglycans to form hydrodynamically related glycocalyx (104), the loss of sdc1 induces a pro-inflammatory endothelial phenotype (105). The breakdown of physical barrier caused by the degradation of glycocalyx leads to the contact of endothelial cells with blood cells and other harmful components, evoking local inflammation, edema, platelet aggregation, oxidative stress, and loss of vascular reactivity (106, 107). In the nervous system, the degradation of endothelial glycocalyx increases the BBB permeability, promotes cerebral edema and impairs vasodilation. Previous studies revealed blood-brain membrane leakage and brain edema upon glycocalyx degradation after asphyxia, cardiac arrest and cardiopulmonary resuscitation in rats (108). Therefore, the repair of glycocalyx has emerged as a potential therapeutic target for multiple brain dysfunctions with BBB breakdown.

Organ or whole body ischemia followed by reperfusion, as found in cardiopulmonary bypass, repair of aortic aneurysms and deep hypothermic cardiac arrest, consistently give rise to elevated levels of sdc1 and heparan sulphate in blood (109). Cardiac arrest syndrome has also been associated with increased plasma syndecan, heparan sulphate and hyaluronan (110). Such phenomena presumably reflect shedding of the endothelial glycocalyx and may account for the development of edema and exacerbated leukocyte and platelet adhesion in reperfused tissue, which contributed to reperfusion damage (111–114). The mediators of ischemia and hypoxia-induced endodermis shedding may be adenosine and inosine, both of which are produced in large quantities by degradation of high-energy adenine nucleotides (ATP, ADP) under hypoxia. Both are stimulants of adenosine type 3 receptors found on human mast cells (115, 116). At least those resident mast cells found in the human myocardium contain granular stores of the enzyme heparanase, release of which into the extracellular space will cause cleavage of heparan sulphate from the endothelial glycocalyx (117, 118). Mast cells also contain a large number of proteases cytokines and chemokines, and potential inducers of sinticon and hyaluronan sheddin (116, 119–121).

The relationship between glycocalyx destruction and disease may be most obvious in sepsis. An increase in plasma sdc1 concentration in individual patients is highly negatively correlated with survival (122–124). Similarly, serum hyaluronan concentration in critically ill patients, most of whom were sepsis patients, depend on the severity of the disease (125). In another study, anti-tumor necrosis factor -α (TNF-α) was used antibody Etanercide injection of lipopolysaccharide reduced the shedding of glycocalyx components in human volunteers (126). Experiments on isolated heart preparations have revealed massive destruction of the glycocalyx after application of TNF-α (119).

Respiratory failure associated with sepsis is accompanied by higher heparanase activity in blood and lung tissue than in normal human biopsies and by elevated plasma heparan sulphate and hyaluronan levels (127, 128). In a mouse model of sepsis, shedding of lung glycocalyx heparan sulphate was induced by lipopolysaccharide (LPS) *via* TNF-α. The authors speculate that this occurs through activation of endothelial heparinase. Subsequent lung microvascular degradation of glycocalyx promotes adhesion of neutrophils (PMN) (127). PMN contains many proteolytic enzymes, including serine protease elastase and protease 3. These enzymes may explain the cleavage of the syndecans protein core and the shedding of hyaluronan by proteolysis of hyaluronan binding receptor CD44 (129, 130). To determine whether elastin is released from neutrophils attached to the vascular wall, Bernhard F. Pecker et al. injected human neutrophils prestimulated by fMLP into the isolated coronary artery system of guinea pig hear and stained them with anti-human elastin antibodies. Immuno-histochemical examination revealed elastase, both in granula within the PMN and beginning to spread out from the PMN along the surface of the endothelial vessel lining, then into the glycocalyx (102).

## Heparin Ameliorates BBB Dysfunction by Regulating Glycocalyx

Heparin is a type of glycosaminoglycan with anticoagulant and anti-inflammatory features that has been widely used in clinical practice to prevent venous thromboembolism (VTE). Glycosaminoglycan belongs to glycocalyx in essence, so the heparin acts as an exogenous glycosaminoglycan to repair or protect glycocalyx (131). Recently, some animal studies and clinical trials have revealed other benefits of heparin in addition to its anticoagulant function, including protease modulation, anti-complement and anti-inflammatory activities (132, 133). Moreover, heparin regulates angiogenesis (134), leucocyte recruitment (135), platelet activation (136), adhesion molecule expression (137), and cytokine release stimulated by lipopolysaccharide (LPS) (93). Yini et al. observed a reduction of glycocalyx shedding by therapeutic doses of heparin *via* inhibited inflammation in a canine septic shock model (138), which might be due to the inhibition of heparinase. In sepsis, heparin protects glycocalyx from degradation by inhibiting HPSE and the subsequent decomposition of heparin sulfate. A preclinical study reported that the thinning of the glucose calyx layer in pulmonary microvessels was caused by the degradation of heparin sulfate upon TNF-α-dependent heparinase activation, which was weakened by heparin treatment (127). As heparinase promotes MMP expression, heparin, by inhibiting heparinase activity, also reduces MMP levels (139).

The efficacy of heparin in treating BBB dysfunction has also been previously reported. In subarachnoid hemorrhage (SAH), heparin infusion showed benefits for prognosis (13, 14). In preclinical models of SAH, heparin infusion reduced the inflammatory response in brain tissue (14), which might be ascribed to the reduction of leukocyte extravasation (140). Simard JM et al. made rat models of SAH in the experiment. The rats were implanted with mini-osmotic pumps that delivered either vehicle or unfractionated heparin (10 U/kg/h IV) beginning 12 h after SAH. The result showed that administration of heparin significantly reduced neuroinflammation, demyelination, and transsynaptic apoptosis (14). Similarly, James RFet alretrospectively analyzed all patients treated with aSAH between July 2009 and April 2014. In this study, the Montreal Cognitive Assessment (MoCA) was used to evaluate cognitive changes in aSAH patients treated with the Maryland LDIVH protocol compared with controls. This study suggests that the Maryland LDIVH protocol may improve cognitive outcomes in aSAH patients. But a randomized controlled trial is needed to determine the safety and potential benefit of unfractionated heparin in aSAH patients (13).

Brain injury accounts for death or disability from trauma, with persistent tissue inflammation and neurological impairment. This would lead to poor outcomes, including cognitive impairment, paralysis, coma, and brain death. Persistent post-injury inflammation is thought to be the result of BBB breakdown, with massive fluid and cell leakage into the stroma causing cerebral edema. Circulating leukocytes interact with endothelial cells in microcirculation (141, 142), and then cause the release of toxic substances (143–145). Animal studies showed that the early repeated administration of heparin after TBI reduced the contact between active leukocytes and endothelium in the peripheral microcirculation (145), which also diminished the local venular albumin leakage (144). Another study analyzed patients with severe TBI admitted to a level 1 trauma center in 2009-2010. These patients were classified into one of three groups. Those who received the first dose of prophylactic subcutaneous heparin or LMWH in the first 72 hours after admission (early) or 5 days or more after admission (late). The others receiving initial prophylactic heparin analogs after 72h and before 120h after admission were classified into the intermediate group. The results of this study indicate that the slowest progression of brain injury on repeated head CT scans was in the early group up to 10 days after admission. It suggested that early administration of heparin in patients with severe TBI improved patient prognosis (15). In the case of status epilepticus (SE), animal epilepsy models showed that BBB damage allows leukocytes, cytokines, chemokines and fluids to enter the brain parenchyma when the glycocalyx is degraded. Astrocytes and microglia were activated, which aggravated the inflammatory response and tissue edema in the brain and further damaged the BBB, forming a vicious cycle. In this experiment, the mice treated with heparin showed less degradation of glycocalyx after SE compared with the control group. It suggested the importance of glycocalyx degradation in cerebral edema and SE outcome, and indicated heparin treatment might be a new strategy for brain protection in SE (16).

## Discussion

Normal neurological function depends on various balance machinery inside brain, particularly regulation of the BBB. Endothelial glycocalyx acts as an important component affecting BBB function. Notably, endothelial glycocalyx is susceptible to damage, resulting in the BBB breakdown and the aggregation of brain injury. Restoration or maintenance of the structure and function of endothelial glycocalyx is a promising therapeutic strategy for various brain disorders. Therefore, studies on the relationship between the endothelial glycocalyx and the integrity of BBB have drawn much attention. The glycocalyx of the vascular endothelial cells of the nervous system is thicker than that of the heart and pulmonary. However, no study has been conducted to explain the significance of these two different phenomena. In sepsis and other systemic diseases, whether the nervous system is less vulnerable to damage due to thicker glycocalyx. It is also the direction of our next research. Heparin has shown considerable potency for the protection of glycocalyx, which alleviates the manifestation and improves the prognosis of neurological diseases associated with BBB dysfunction. But at present, there are few studies on the treatment of BBB with heparin, especially clinical studies. Clinical studies have shown improvement in brain function through clinical scores and imaging. Animal experiments also demonstrated the therapeutic effect of heparin through parameter measurement. These studies cannot fully suggest that heparin has a positive effect on glycocalyx integrity or BBB functionality. Moreover, heparin itself is an anticoagulant substance, in order to ensure the safety of patients with subarachnoid hemorrhage, a large number of clinical trials are still needed to study the complications of using heparin in such patients. The glycocalyx acts as part of the blood-brain barrier, and even more as part of the nervous system. In turn, the nervous system as a whole has all its components connected to maintain its normal function. In the future, more basic and clinical studies on the extensive mechanism of heparin to maintain BBB integrity by protecting glycocalyx are warranted.

## Author Contributions

MC, XL, and JZ: collected and analyzed the data. JZ and RY: drafted the article. XZ: revised and submitted the manuscript. All authors have read and approved the final version of manuscript.

## Funding

The present review was supported by the Liaoning Natural Science Foundation (grant No. 2021-MS-188)

## Conflict of Interest

The authors declare that the research was conducted in the absence of any commercial or financial relationships that could be construed as a potential conflict of interest.

## Publisher’s Note

All claims expressed in this article are solely those of the authors and do not necessarily represent those of their affiliated organizations, or those of the publisher, the editors and the reviewers. Any product that may be evaluated in this article, or claim that may be made by its manufacturer, is not guaranteed or endorsed by the publisher.
